# Chitosan/Zeolite Composite Aerogels for a Fast and Effective Removal of Both Anionic and Cationic Dyes from Water

**DOI:** 10.3390/polym13111691

**Published:** 2021-05-22

**Authors:** Angela Marotta, Enrica Luzzi, Martina Salzano de Luna, Paolo Aprea, Veronica Ambrogi, Giovanni Filippone

**Affiliations:** 1 Dipartimento di Ingegneria Chimica, dei Materiali e della Produzione Industriale, Università di Napoli Federico II, 80125 Naples, Italy; e.luzzi@studenti.unina.it (E.L.); martina.salzanodeluna@unina.it (M.S.d.L.); paolo.aprea@unina.it (P.A.); ambrogi@unina.it (V.A.); 2 INSTM Consortium, Via Giusti 9, 50125 Firenze, Italy; 3 Institute for Polymers, Composites and Biomaterials (IPCB)–CNR, Via Campi Flegrei 34, 80078 Pozzuoli, Italy

**Keywords:** chitosan, zeolite 13X, aerogel, dye adsorption, broad-spectrum ability, mechanical properties, reusability

## Abstract

Organic dyes are extensively used in many industrial sectors, and their uncontrolled disposal into wastewaters raises serious concerns for environmental and human health. Due to the large variety of such pollutants, an effective remediation strategy should be characterized by a broad-spectrum efficacy. A promising strategy is represented by the combination of different adsorbent materials with complementary functionalities to develop composite materials that are expected to remove different contaminants. In the present work, a broad-spectrum adsorbent was developed by embedding zeolite 13X powder (ZX) in a chitosan (CS) aerogel (1:1 by weight). The CS–ZX composite adsorbent removes both anionic (indigo carmine, IC) and cationic (methylene blue, MB) dyes effectively, with a maximum uptake capacity of 221 mg/g and 108 mg/g, respectively. In addition, the adsorption kinetics are rather fast, with equilibrium conditions attained in less than 2 h. The composite exhibits good mechanical properties in both dry and wet state, which enables its handling for reusability purposes. In this regard, preliminary tests show that the full restoration of the IC removal ability over three adsorption–desorption cycles is achieved using a 0.1 M NaOH aqueous solution, while a 1 M NaCl aqueous solution allows one to preserve >60% of the MB removal ability.

## 1. Introduction

Wastewaters containing leftovers of organic dyes from several industrial processes are often discharged into the environment [1]. The textile industry is responsible for more than half of the effluents, releasing around 100 tons of dyes per year into environmental water bodies [1]. A wide number of hazardous chemicals are necessary to ensure color-giving properties, making these pollutants stable to degradation and water soluble, but also toxic for aquatic life and dangerous for environmental and human health [1,2]. In this scenario, the development of remediation strategies is crucial to reduce water contamination and to eliminate the environmental impact of wastewater disposal. For this purpose, physical, chemical and biological methods have been widely investigated, e.g., chemical precipitation, enzymatic decomposition, membrane filtration, and adsorption.

Despite being a simple and efficient processes, chemical methods such as precipitation, coagulation and oxidation are disadvantaged by the consumption of high-cost chemicals and high sludge production [3,4]. Regarding the possible enzymatic routes, many microorganisms can degrade several pollutants, but their employment is often limited by technological constraint and reduced flexibility of the process [4,5]. Membrane filtration-based processes are commonly considered as very efficient even at high concentration, but the typical production of sludge requires frequent membrane replacement [6,7,8]. Adsorption is an easy, cost-effective and non-selective method suitable for the removal of a wide range of pollutants. The working principle is the exploitation of an active material able to adsorb specific chemical species [9]. Polymer-based foams or aerogels based on cellulose [10,11], alginate [12], and chitosan (CS) [13,14,15,16] have been extensively explored for water remediation purposes. High surface area nanoparticles [17,18] and micro/meso-porous particles, such as zeolites and metal–organic frameworks [4,19,20,21,22,23] have also been widely investigated as effective adsorbents. However, their reuse is hindered by the difficult recovery from polluted solutions, that can often lead to secondary pollution. Conversely, monolithic adsorbents, such as aerogels, are easier to handle, allowing for simple and effective regeneration processes. Whatever its nature, an ideal adsorbent material should be able to remove high amounts of different dyes. Since the latter can differ very much in terms of chemical/physical features, the development of effective broad-spectrum adsorbents can be very challenging. A first obstacle is the surface charge of the adsorbent, which must match that of the molecules to be removed: positively charged adsorbents are suited for the removal of anionic dyes, but they are much less effective towards cationic species. As a result, most of the adsorbents developed so far are not able to simultaneously remove both anionic and cationic dyes. A possible approach to overcome this limitation is combining two adsorbent materials with complementary dye removal capacity into a broad-spectrum two-phase adsorbent. Following this principle, remarkable results were obtained by dispersing carbonaceous fillers, effective in removing cationic dyes, or magnetic nanoparticles, in a CS framework, which is instead effective towards anionic pollutants [18,24,25,26]. In another recent study, CS was mixed with alginate, which exhibits high affinity towards cationic dyes [27].

Here, a novel composites adsorbent based on zeolite 13X (ZX) and CS was developed to achieve broad-spectrum adsorption capacity. High porosity and enhanced surface charge provides ZX with optimal adsorption capacity towards cationic dyes [4]. In principle, the low ability of ZX to capture anionic dyes can be improved by amine-functionalization [28,29,30], but the resulting powder is not easy to handle in practical applications, where monolithic adsorbents are preferable. Besides providing complementary dye removal ability towards anionic species, CS was also selected here to impart mechanical strength and coherence to ZX powder [31]. Composite CS–ZX aerogels were prepared by freeze-drying to induce high macroporosity to the CS phase, so as not to occlude the inherent porosity of ZX. This led to an optimal combination of the complementary adsorption capacities of the two components, as proved by isothermal and kinetics experiments. Indigo carmine (IC) and methylene blue (MB) were used as anionic and cationic dye probes, respectively. A thorough mechanical characterization of the adsorbents was performed in both dry and wet conditions to assess the mechanical resistance of the composite aerogels, which is crucial for reusability purposes. The latter was also verified by regenerating and testing again the dye removal abilities of the samples over three adsorption/desorption cycles.

## 2. Materials and Methods

### 2.1. Materials

Chitosan powder (molecular weight 190–310 kDa, deacetylation degree 75–85%), Glutaraldehyde (GLA) 25% aqueous solution, zeolite FAU 13X (average particle size 2 µm) and acetic acid were all supplied by Sigma-Aldrich and used as received. Indigo carmine (dye content ≥ 85%) was purchased from Carlo Erba and methylene blue (dye content ≥ 82%) from Sigma-Aldrich.

### 2.2. Aerogels Preparation

CS powder (20 mg mL^−1^) was dissolved in acetic acid aqueous solution (2 vol.%) under stirring at room temperature for 72 h. The pH of the CS solution was increased from ~3.5 up to ~5.1 by adding dropwise a proper amount of 1M NaOH aqueous solution. ZX in water dispersion was added to the CS solution to obtain a CS–ZX mixture (1:1 by weight). GLA (1 wt.% with respect to CS) was added under vigorous stirring for 30 s, and the resulting mixture was poured into cylindrical plastic molds and rapidly frozen by immersion in acetone at −30 °C. Macroporous aerogels were obtained by lyophilization. The latter were thermally treated at 90 °C for 1.5 h to ensure complete crosslinking according to the procedure reported in a previous work [25], and verifying the absence of unreacted GLA by Fourier transform infrared spectroscopy (see Figure S1 in Supplementary Materials). The samples were subsequently washed with bi-distilled water to remove the sodium acetate possibly formed during the process [31]. A second freeze-drying step was finally performed to obtain the CS–ZX composite aerogels. Except for the step consisting in the addition of the ZX solution, the same procedure was adopted to prepare pristine CS aerogels.

### 2.3. Characterization

The inner microstructure of the aerogels was inspected by Scanning Electron Microscopy (SEM, TM3000 by Hitachi). The samples were razor blade-cut and sputtered with gold prior to observations.

The ZX stability during the sample preparations was proved by XRD analyses, carried out using a Malvern Panalytical X’Pert Pro X-ray diffractometer equipped with a PIXcel1D detector. The 2θ scan range was 5–60° with a step size of 0.01°.

The dye equilibrium adsorption capacity (*q_e_*, mg g^−1^) of the ZX powder and of the CS and CS–ZX aerogels was tested through equilibrium batch adsorption experiments. MB and IC mono-component solutions at different concentrations (C_0_, mg L^−1^) ranging from 50 to 750 mg L^−1^ were obtained by diluting stock solutions. The same proper amount of each solution was added to the same quantity of CS, ZX and CS–ZX samples. Isothermal adsorption conditions were reached by shaking the adsorbent–dye solution with a Shaker Incubator SKI 4 Argo Lab at 150 rpm and T  =  25 °C until equilibrium with no pH adjustment. The equilibrium dye concentration (C_e_, mg L^–1^) was measured by UV analysis at the characteristic maximum absorbance wavelength of each dye (611 nm for IC, 665 for MB) with an ONDA UV–Vis spectrophotometer, equipped with VL-4LC UV-light source (8 W, 365 nm). The examined wavelength range was 550–700 nm. The following Equation allowed the determination of *q_e_*:

(1) $$q_{e}=\frac{\left(C_{0}-C_{e}\right)V}{m}$$

where *m* is the mass of the adsorbent sample and *V* is the volume of the dye solution.

Kinetic adsorption tests were performed at the same *C*_0_ of 200 mg L^–1^ for both IC and MB with CS–ZX samples. Aliquots of the dye solutions were collected at predetermined time intervals and the dye concentration was assessed by UV analysis. The amount of dye (*q_t_*, mg g^–1^) at any time interval was calculated as follows:

(2) $$q_{t}=\frac{\left(C_{0}-C_{t}\right)V}{m}$$

where *C_t_* (mg L^–1^) is the dye concentration at any time, *m* is the mass of the adsorbent sample and *V* is the volume of the dye solution. All the adsorption tests were repeated three times on different samples obtained from distinct preparation cycles.

The CS and CS–ZX aerogel swelling degree (SD) was calculated from the fully swollen weight change before and after immersion in bi-distilled water, according to the following Equation:

(3) $$SD=\frac{\left(W_{s}-W_{d}\right)}{W_{d}}100\%$$

where *W_s_* is the weight of the fully swollen aerogel, and *W_d_* is the dry aerogel weight. Data presented are average values evaluated on three measurements on different samples.

The apparent density of the aerogels was obtained as the ratio between the samples’ weight and their equivalent volume, defined as the volume of the cylinder with the same diameter and height as the aerogel samples, of ten distinctly prepared samples.

The aerogel mechanical properties were investigated by uniaxial compression/tensile tests (Tensometer 2020) performed at 1 mm min^–1^ on unconfined cylindrical-shaped samples (average diameter of 11 mm and average height of 7 mm). Tests were conducted in both dry and wet conditions, recording the signals during loading–unloading cycles. Wet tests were performed by immersing the samples in bi-distilled water, and the recorded forces were corrected to account for buoyant forces. Three experiments were replicated for each condition. The compressive modulus was calculated as the slope of the initial linear portion of the stress–strain curve during loading.

The possibility of reusing CS–ZX aerogels was assessed by performing three adsorption/desorption cycles on the same sample at an initial dye concentration of 200 mg mL^–1^. The equilibrium dye concentration was evaluated with UV analysis by using Equation (1). The samples were regenerated by immersion in for 72 h in 0.1 M and 1 M solutions of NaOH (for IC) and NaCl (for MB). A regeneration efficiency (R) was defined as the ratio between *q_e_* calculated at each adsorption cycle and that evaluated during the first adsorption cycle.

## 3. Results and Discussion

**Figure 1:**
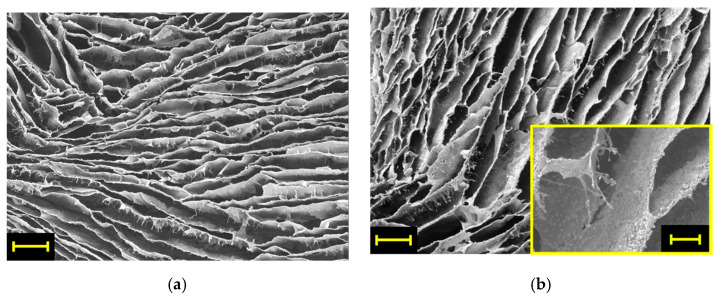
Representative SEM micrographs of (**a**) CS and (**b**) CS–ZX aerogels. Scale bar is 200 µm (the bar in the inset of (**b**) is 50 µm).

The microstructure of the aerogels is shown in Figure 1, where representative SEM micrographs of pristine CS and CS–ZX samples are reported. The cylindric-shaped molds induce a radial growth of the ice crystals during sample freezing, which eventually results in an anisotropic open porous structure with the chitosan walls retracing the former ice crystals. The two samples share comparable morphology at the micron scale, in which lamellar pores are separated by very fine polymeric walls. Although the aspect ratio of the pores is slightly reduced upon ZX addition, the structure of the CS–ZX aerogels is still made of highly elongated porous channels which are expected to facilitate the access of contaminated water to the active sites on the inner parts of the adsorbent [14]. Regarding the dispersion of ZX, the particles appear homogeneously dispersed within the CS walls (see inset of Figure 1b).

**Figure 2:**
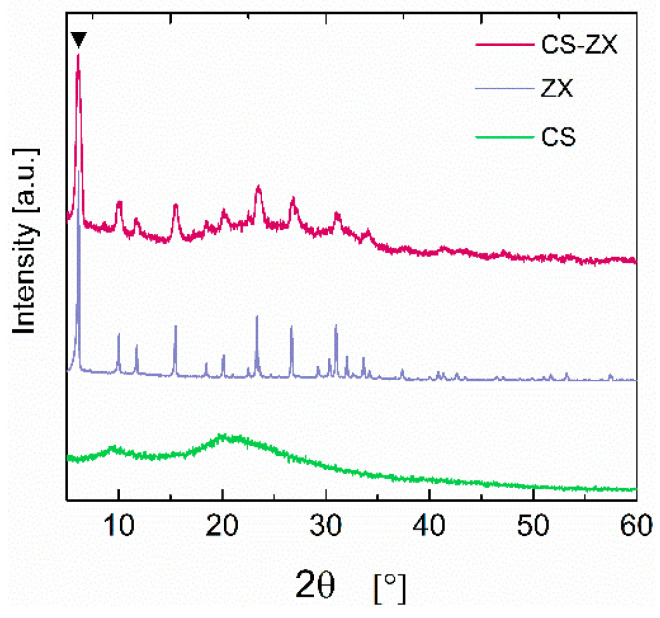
XRD patterns of ZX, CS and CS–ZX samples. Peak at 2θ = 6.228° is characteristic of zeolite 13X.

To verify that the preparation protocol did not damage the ZX crystalline structure, XRD analyses were performed. As shown in Figure 2, the zeolite 13X characteristic peaks (e.g., 2θ = 6.228°) emerge in the CS–ZX pattern, proving that the pH adjustment with NaOH solution is sufficient to prevent ZX from structural changes. This is an important point, as the adsorption capacity of zeolites is strictly related to their inherently crystalline structure. Moreover, XRD patterns also indicate the complete removal of sodium acetate possibly formed during the aerogel preparation, as revealed by the absence of its characteristic peaks at 2θ = 8.88°, 27.208° and 30.95°.

### 3.1. Dye Ddsorption

**Figure 3:**
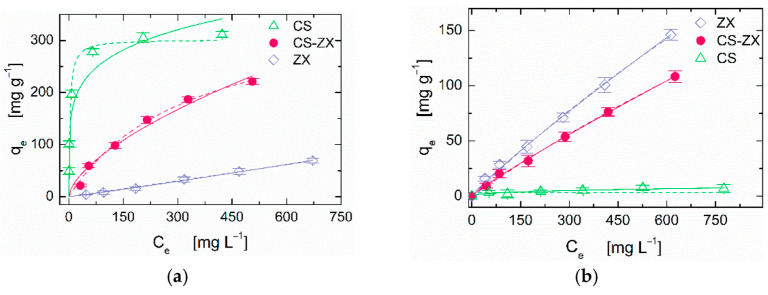
(**a**) IC and (**b**) MB adsorption isotherms of ZX powder and CS, CS–ZX aerogels. Solid lines and dashed lines represent the best fitting of the Freundlich and Langmuir models to the experimental data, respectively (see Section S1 of Supplementary Material for details).

The broad-spectrum adsorption ability of CS–ZX aerogels was tested using IC and MB as model molecules for anionic and cationic pollutants, respectively. The equilibrium adsorption isotherms of ZX particles, pristine CS and composite CS–ZX aerogels towards IC and MB dyes are reported in Figure 3.

The adsorption behavior of all systems can be satisfactorily described by both the Freundlich and Langmuir model [32], but the latter better fits the experimental data (see Section S2 in Supporting Information) and can be applied to give a reliable estimation of the maximum adsorption capacity [33]. First, we discuss the performance of the individual constituents. As expected, pristine CS aerogel exhibits excellent ability in binding the anionic IC dye thanks to protonated amino groups on the CS chains, but it is ineffective for cationic species, such as MB, due to unfavorable electrostatic interactions [34]. ZX particles show the opposite behavior, as their negatively charged surface mostly attracts the positively charged groups of cationic dyes [35]. The non-negligible adsorption of IC observed for ZX powder can be explained by the hydrogen bonding that occurs between nitrogen atoms of the dye and silanol group of zeolite [35]. The complementary features of CS and ZX effectively combine in the composite aerogel, providing the CS–ZX sample with good adsorption ability towards both IC and MB. The maximum adsorption capacity experimentally accessed through isotherm tests was about 221 mg g^−1^ and 108 mg g^−1^ at C_0_ = 750 mg L^−1^ for IC and MB, respectively. The theoretical values predicted by Langmuir model are even higher, reaching limiting values of 372.83 ± 29.15 mg g^−1^ for IC and 7058.95 ± 208.39 mg g^−1^ for MB.

Zeolite-based adsorbents have been mostly considered for cationic dyes removal due to their negative surface charge. MB adsorption onto nanosheet MFI zeolite has been reported to reach a remarkable maximum adsorption capacity of about 195 mg g^−1^ under a C_0_ of 250 mg L^−1^, thanks to an optimization of the test conditions by adjusting the dye solution pH to 10 [20]. The influence of pH value and adsorption test conditions were also investigated for zeolite 4A applied for the same purposes, obtaining a predicted maximum adsorption capacity of about 44.35 mg g^−1^ under a C_0_ of 50 mg L^−1^ [19]. Zeolite 13X synthesis was optimized for MB removal purposes, achieving a maximum adsorption capacity of about 144.56 mg g^−1^ under a C_0_ of 50 mg L^−1^ [35]. Nevertheless, similar remarkable performances are difficult to achieve when zeolites are applied in anionic dyes removal. Mesoporous ZSM-5 zeolite exploited for methyl orange removal exhibits a maximum adsorption ability of only 4.71 mg g^−1^ [36]. Embedding ZSM-5 in a sodium alginate-based membrane allows for a significant improvement in the performance, leading to *q_e_* of 71.76 mg g^−1^, but such a notable result was achieved without extending the removal ability to cationic pollutants [37]. Similarly, other polymer-based systems such as hyperbranched polyamide functionalized cellulose [38], or chitosan-benzil-ZnO-Fe_3_O_4_ nanocomposite [18] have proved to possess excellent abilities in anionic dyes removal (above 800 mg g^−1^ for orange II and 570.8 mg g^−1^ for Remazol Brilliant Blue R, respectively), but the removal cationic dyes was not assessed. In our samples, such a capacity is provided by CS, making our CS–ZX composite aerogels an effective solution for wide-spectrum dye removal.

**Figure 4:**
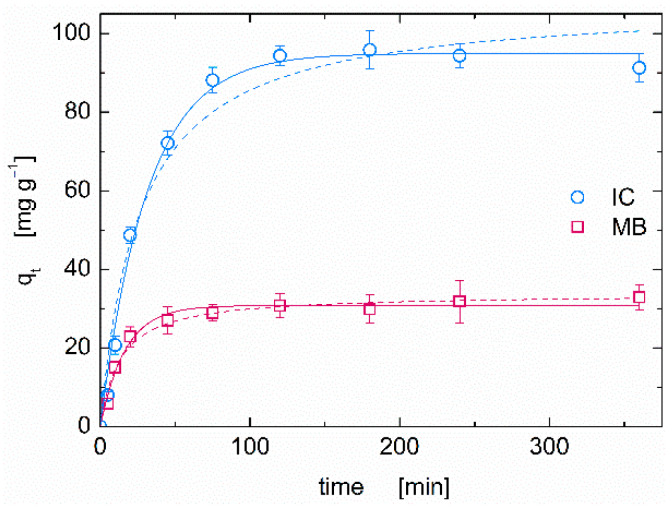
IC and MB adsorption kinetics of CS–ZX aerogels at C_0_ = 200 mg L^−1^. Solid lines and dashed lines represent the best fitting of the Pseudo First Order and Pseudo Second Order models to experimental data, respectively (see Section S1 of Supplementary Material for details).

The removal kinetics for IC and MB from aqueous solution using CS–ZX aerogels was also investigated, as it represents a crucial aspect in the development of new adsorbents. The amount of removed dye (*q_t_*, see Equation (2)) at C_0_ = 200 mg L^−1^ is reported as a function of time in Figure 4. The initial adsorption step is very rapid for both the cationic and anionic dye, as a large number of active sites for both constituents are available in the earlier stages of the adsorption process [27]. Then, the kinetics slows down until equilibrium conditions are attained. Non-linear Pseudo-First-Order (PFO) and non-linear Pseudo-Second-Order (PSO) models are used to fit the systems behavior [39]. PFO exhibits a better accordance with the experimental data (Section S2 in Supporting Information). Referring to the PFO model, the time required to attain 95% of the sample adsorption saturation (t_eq_, equilibrium time) was t_eq_ = 95 min for IC and 50 min for MB. The limiting values of *q_t_* at C_e_ = 200 mg L^−1^, attained in about 2 h, are 93.97 ± 1.91 mg g^−1^ for IC and 31.31 ± 1.30 mg g^−1^ for MB. A similar behavior has been observed in hyperbranched polyamide functionalized cellulose applied for anionic orange II removal [38]. Here, the first rapid step lasts 30 min, reaching 92% of the sample adsorption saturation; then, equilibrium conditions are achieved in 60 min.

The adsorption kinetics of the CS–ZX composite aerogels are much faster than those of similar wide-spectrum chitosan-based systems. Equilibrium times of 58 h and 36 h were reported to remove MB and anionic eosin Y with graphene oxide–chitosan hydrogels [24], while ~10 h and ~100 h were necessary for MB and anionic acid black-172 using a chitosan–alginate composite foam [27]. Graphene oxide powder can be used to go down to a t_eq_ of ~10^2^ min [40], but the price to pay is dealing with an incoherent adsorbent. Differently, as discussed in the next Section, our CS–ZX composite aerogels are easy to handle, and this is an undoubted advantage for reusability purposes.

### 3.2. Mechanical Properties

**Figure 5:**
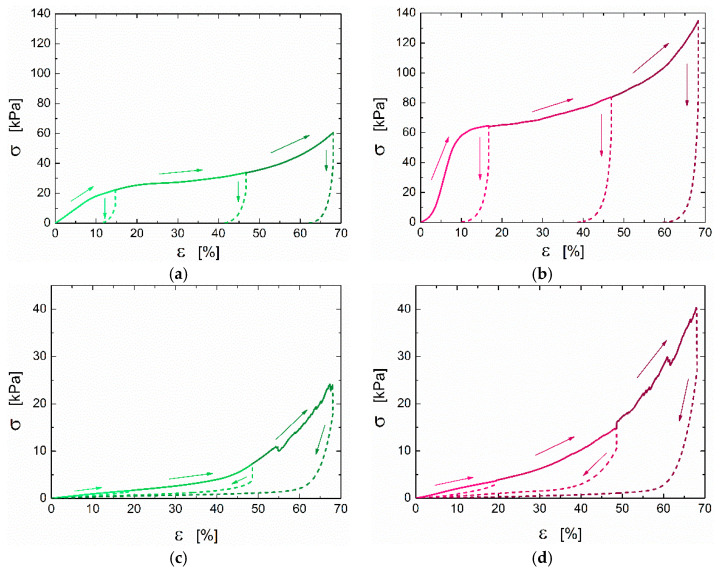
Representative compression stress–strain curves for CS in dry (**a**) and wet (**c**) conditions and for CS–ZX in dry (**b**) and wet (**d**) conditions. The arrows indicate loading (continuous lines) and unloading (dashed lines) cycles carried out by setting different values of maximum deformation (20%, 50% and 70%). Each loading–unloading cycle was performed on a distinct sample.

The mechanical behavior of the CS and CS/ZX aerogels under compression was studied not only in dry conditions, but also in the wet state (fully swollen) to mimic the real application scenario. Loading–unloading cycles were performed by setting three different values of maximum strain (20%, 50% and 70%). Concerning the dry state (Figure 5a,b), the aerogels show the typical behavior of cellular structures under compression: at small strain (<10%) they exhibit linear elastic behavior, followed by a plateau at intermediate strain (up to about 40%) and a final densification region at high strains, with a consequent increase in the stress. The elastic compressive modulus of the CS aerogel is one order of magnitude lower than that reported in a previous work [25], which was about 0.35 MPa. This is not unexpected, as here, the crosslinking density was deliberately kept low to preserve the amino groups’ functionality necessary for anionic dye removal. The compressive elastic moduli in both dry and wet conditions are summarized in Table 1. Dry samples are obviously stiffer than wet ones, whose softness reflects the swollen state of the CS. Regarding the effect of ZX, the E_c_ values of the composite are much higher than those of the pure CS sample, especially in the dry state. Even normalizing the moduli over the sample densities, the CS/ZX sample confirms to be stiffer than pure CS. This is an indirect confirmation of the good dispersion and embedding of the ZX particles in the CS matrix emerged from the visual inspection of the SEM micrographs. Particles with poor interfacial adhesion, in fact, would have a detrimental effect on the mechanical properties [41]. Finally, looking at the unloading cycles, the dry samples remain flattened after compression, recovering less than 10% of deformation when the load is removed. Differently, the samples almost fully recover compressive deformation in wet conditions. This allows for a simple recovery of the swollen samples for regeneration purposes.

### 3.3. Aerogels Reusability

The reusability of the CS–ZX aerogels was assessed by repeating three adsorption experiments on the same sample after its regeneration. The latter step aims at restoring the adsorption capacity by inducing the release of the dye adsorbed in previous cycle(s). NaOH and NaCl aqueous solutions were used to desorb IC an MB, respectively. NaOH and NaCl were selected for their ability to act on sites responsible for the adsorption of chitosan and zeolite, respectively. In particular, NaOH is expected to protonate the amino groups of chitosan, while NaCl is expected to influence the charge distribution on the zeolite surface, thus inducing the desorption of the dye molecules.

**Figure 6:**
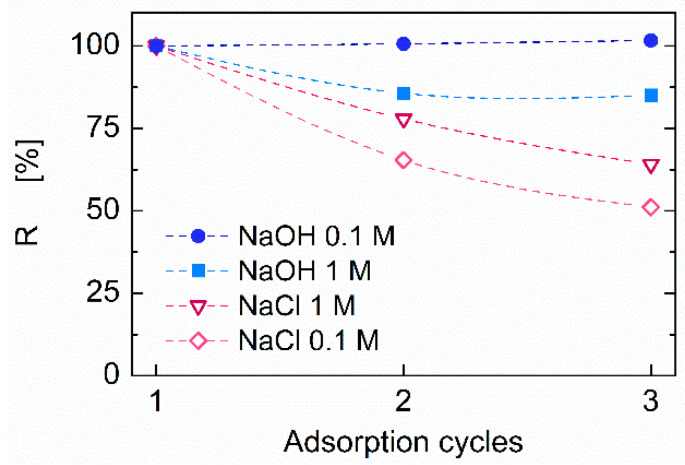
Cyclic adsorption capacity of CS–ZX aerogels for IC (full symbols) and MB (open symbols). Dye concentration of each adsorption cycle was C_0_ = 200 mg L^−1^.

The results of the reusability tests are shown in Figure 6, where the regeneration efficacy (R) is reported as a function of the number of adsorption–desorption cycles.

Complete IC release was successfully achieved with a NaOH 0.1 M solution, and the regenerated sample exhibits the same performance as the virgin one over three cycles. A slight decrease in R is noticed when a more concentrated 1 M NaOH solution is used. This is likely due to some interference with the protonated amino groups of CS in excessively alkaline conditions. Differently, the complete desorption of MB is not achieved with the NaCl solution. This leads to a gradual decrease in R down to 60% or 50% after three adsorption cycles, depending on the solution molarity. This loss in adsorption capacity can be attributed to an incomplete desorption of the pollutant entrapped in the ZX porosity during previous adsorption cycle(s). The consequent reduction in available sites where MB molecules can be entrapped results in a decreasing adsorption capacity. Better results could be achieved by air calcination at about 500 °C, which has been proved to be an effective protocol to regenerate natural zeolite after MB adsorption [42]. Such a procedure, however, would irremediably damage the CS phase, which is not stable at such high temperatures [31]. Preserving a though reduced cationic dye removal ability while maintaining the anionic cationic dye removal ability ensured by CS is preferable. Overall, given their wide-spectrum adsorption capacity and the possibility of reusing the samples without excessive losses in performance, our CS–ZX composite aerogels can be considered promising systems for dye removal.

## 4. Conclusions

Broad-spectrum adsorbents for the effective removal of both cationic and anionic dyes from water were successfully produced by dispersing zeolite 13X in a chitosan-based aerogel. The microporous structure obtained by freeze-drying allows for a fast diffusion of contaminated water to the active sites of the constituents of the composite, which exhibit complementary affinity towards anionic (chitosan) and cationic (zeolite) dyes. This feature results in fast dye removal kinetics (adsorption equilibrium reached in ~2 h) and broad-spectrum adsorption ability (uptake capacity of 221 mg g^−1^ of anionic indigo carmine and 108 mg g^−1^ of cationic methylene blue). The samples also possess adequate mechanical strength to be handled in both wet and dry conditions for reuse purposes. Substantial restoration of the dye removal ability was obtained by using NaOH or NaCl water solutions, as proved over three consecutive adsorption–desorption cycles performed on the same sample. Overall, our results prove that the addition of zeolite 13X to chitosan aerogels is a viable solution to widen the still meager range of wide-spectrum and reusable adsorbent materials for dye removal.

## Figures and Tables

**Table 1:** Values of compressive elastic modulus, specific compressive elastic modulus density and swelling degree.

Sample	E_C_ [kPa]		ρ [g cm^−3^]	E_C_/ρ [kPa·g^−1^ cm^−3^]		SD [%]
CS	dry	2.25 ± 0.67	0.022 ± 0.001	dry	103.7	47.5 ± 1.9
	wet	0.13 ± 0.07		wet	5.8	
CS–ZX	dry	13.13 ± 8.63	0.043 ± 0.003	dry	305.6	23.0 ± 2.9
	wet	0.23 ± 0.07		wet	5.3	

## Data Availability

Not applicable.

## Supplementary Materials

The following are available at https://www.mdpi.com/article/10.3390/polym13111691/s1. Figure S1: FTIR spectra of CS and CS-ZX aerogels, Figure S2: TG curves of CS-ZX aerogels, Table S1: Langmuir and Freundlich model param-eters for all the investigated systems, Table S2: Kinetic plots fitting parameters for CS-ZX aerogels.
