# Supplementary material for: Chitosan/Zeolite Composite Aerogels for a Fast and Effective Removal of Both Anionic and Cationic Dyes from Water
Source: Polymers (Basel). 2021 May 22;13(11):1691. doi: 10.3390/polym13111691 (PMC8196880; doi:10.3390/polym13111691)
Supplement: Supplementary file 1 [file polymers-13-01691-s001.zip › polymers-1230178-supplementary.pdf]

polymers

Supporting Information: Chitosan/Zeolite Composite Aerogels for a Fast and Effective Removal of Both Anionic and Cationic Dyes from Water

Angela Marotta, Enrica Luzzi, Martina Salzano de Luna, Paolo Aprea, Veronica Ambrogi, Giovanni Filippone

S1. FTIR analysis

Fourier Transform Infrared (FTIR) analysis was performed in Attenuated Total Reflectance (ATR) mode with a Perkin Elmer Spectrum 3 Delta. The ATR spectra of chitosan and chitosan-zeolite aerogels were recorded at a resolution of 4 cm^{-1} and 4 scan collections and reported in Figure S1.

The spectrum of CS sample is characterized by stretching vibrations of OH and ammine groups (around 3300 cm^{-1}) [1]. Moreover, CH_3 symmetric stretching, C-O-C stretching, and C-OH stretching are visible at around 2920 cm^{-1}, 1150 cm^{-1} and 1065 cm^{-1}, respectively [1]. CS–ZX sample spectrum reflects the composition of the composite, with the compresence of the characteristic bands of zeolite (~750 and 680 cm^{-1}: symmetrical stretching vibration of Al-O-Al and Si-O-Si [2]; ~970 cm^{-1}: Si–O stretching vibrations [3]) and chitosan.

Crosslinking is induced by Glutaraldehyde in both samples, as proved by the presence of the peaks at 1655 cm^{-1} and at 1562 cm^{-1}, that can be addressed to the formation of imine N=C and ethylenic C=C bonds, respectively [1]. Moreover, complete crosslinking is ensured by the absence of unreacted GLA, as highlighted by the absence of free aldehydic groups peak at 1720 cm^{-1} [1].

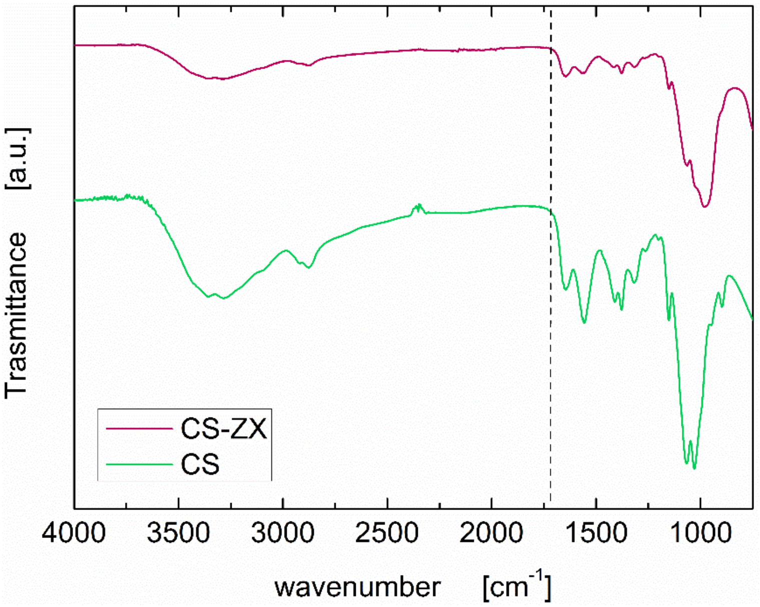

Figure S1. FTIR spectra of CS and CS–ZX aerogels. Baseline correction was applied to the spectra, which are also vertically shifted for the sake of clarity.

S2. Fitting of adsorption isotherms

The adsorption isotherms in Figure 3 of the main text were fitted to the Langmuir and Freundlich models, according to Equation S1 and S2, respectively. The obtained best fitting parameters are reported in Table S1 together with R-squared parameter (RS).

$$q_e = \frac{q_e^{\max} b C_e}{1 + b C_e} \quad \text{(S1)}$$

$$q_e = k C_e^{1/n} \quad \text{(S2)}$$

Table S1. Langmuir and Freundlich model parameters for all the investigated systems.

		IC		
		**CS**	**CS–ZX**	**ZX**
**Langmuir**	q_e^{max} **[mg/g]**	302.27±8.91	372.83±29.15	$3.79 \cdot 10^5 \pm 1.66 \cdot 10^8$
	**b [L/mg]**	0.32±0.06	$2.96 \cdot 10^{-3} \pm 4.41 \cdot 10^{-4}$	$2.69 \cdot 10^{-7} \pm 1.18 \cdot 10^{-4}$
	**RS**	0.99	0.99	0.99
**Freundlich**	**k [L/mg]**	110.65±19.32	4.74±1.65	0.07±0.02
	**n**	5.36±1.02	1.60±1.16	0.93±0.03
	**RS**	0.93	0.99	0.99
		**MB**		
		**CS**	**CS–ZX**	**ZX**
**Langmuir**	q_e^{max} **[mg/g]**	9.20±1.90	7058.95±208.39	850.81±304.32
	**b [L/mg]**	$0.47 \cdot 10^{-3} \pm 0.26 \cdot 10^{-2}$	$2.89 \cdot 10^{-4} \pm 9.74 \cdot 10^{-5}$	$3.35 \cdot 10^{-4} \pm 1.39 \cdot 10^{-4}$
	**RS**	0.86	0.99	0.99
**Freundlich**	**k [L/mg]**	0.49±0.30	0.32±0.05	0.51±0.08
	**n**	1.14±0.03	1.11±0.03	2.44±0.62
	**RS**	0.99	0.99	0.88

The kinetics in Figure 4 of the main text were fitted to the Pseudo First (equation S3) and Pseudo Second Order (Equation S4) and the obtained best fitting parameters are reported in Table S2 together with R-squared parameter.

$$q_t = q_e\left(1 - e^{-k_1 t}\right) \quad \text{(S3)}$$

$$q_t = \frac{q_e^2 k_2 t}{1 + q_e k_2 t} \quad \text{(S4)}$$

Table S2. Kinetic plots fitting parameters for CS–ZX aerogels.

		IC	MB
**Pseudo First Order**	q_e **[mg/g]**	94.96±1.79	30.77±0.69
	k_1 **[s⁻¹]**	0.032±0.002	0.060±0.006
	**RS**	0.99	0.98
**Pseudo Second Order**	q_e **[mg/g]**	107.94±5.37	33.78±0.10
	k_2 **[s⁻¹]**	$3.54 \cdot 10^{-4} \pm 8.78 \cdot 10^{-5}$	$0.002 \pm 3.88 \cdot 10^{-4}$
	**RS**	0.97	0.98

S3. TGA analysis

Thermogravimetric (TG) analyses were carried out using a TGA Q500 (TA Instruments) with a heating rate of 20 °C min-1 in air atmosphere from 25°C to 700°C. CS–ZX samples subjected to the three regeneration cycles have been dried overnight at 60°C under vacuum and then subjected to thermogravimetric analysis. A representative thermogram is reported in Figure S2, together with that of a freshly prepared CS–ZX sample. Apart from the different content of adsorbed water (corresponding to the weight loss up to 200°C), the two curves are comparable, suggesting that the leaching of zeolite after the regeneration cycles is essentially negligible.

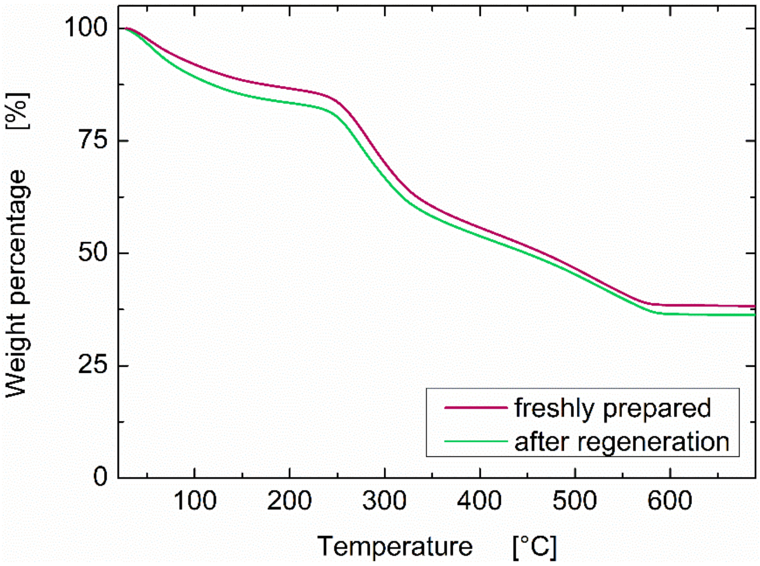

Figure S2. TG curves of CS–ZX aerogels freshly prepared and after three regeneration cycles.

References

1. Salzano de Luna, M.; Ascione, C.; Santillo, C.; Verdolotti, L.; Lavorgna, M.; Buonocore, G.G.; Castaldo, R.; Filippone, G.; Xia, H.; Ambrosio, L. Optimization of dye adsorption capacity and mechanical strength of chitosan aerogels through crosslinking strategy and graphene oxide addition. *Carbohydr. Polym.* **2019**, *211*, 195–203, doi:10.1016/j.carbpol.2019.02.002.
2. Yi, H.; Yang, X.; Tang, X.; Zhao, S.; Wang, J.; Cui, X.; Feng, T.; Ma, Y. Removal of toluene from industrial gas over 13X zeolite supported catalysts by adsorption-plasma catalytic process. *J. Chem. Technol. Biotechnol.* **2017**, *92*, 2276–2286, doi:10.1002/jctb.5314.
3. Safak Boroglu, M.; Ali Gurkaynak, M. Fabrication and characterization of silica modified polyimide-zeolite mixed matrix membranes for gas separation properties. *Polym. Bull.* **2011**, *66*, 463–478, doi:10.1007/s00289-010-0286-x.
